# Functional Analysis in Long-Term Operation of High Power UV-LEDs in Continuous Fluoro-Sensing Systems for Hydrocarbon Pollution

**DOI:** 10.3390/s16030293

**Published:** 2016-02-26

**Authors:** Francisco Jose Arques-Orobon, Neftali Nuñez, Manuel Vazquez, Vicente Gonzalez-Posadas

**Affiliations:** 1 ETSIST—Technical University of Madrid, Madrid 28031, Spain; neftali.nunez@upm.es (N.N.); manuel.vazquez@upm.es (M.V.); vgonzalz@diac.upm.es (V.G.-P.); 2 Instituto de Energía Solar—Technical University of Madrid, Madrid 28040, Spain

**Keywords:** continuous real-time sensing, pollution identification, UV-LED sensing, fluorescence spectroscopy, water pollution

## Abstract

This work analyzes the long-term functionality of HP (High-power) UV-LEDs (Ultraviolet Light Emitting Diodes) as the exciting light source in non-contact, continuous 24/7 real-time fluoro-sensing pollutant identification in inland water. Fluorescence is an effective alternative in the detection and identification of hydrocarbons. The HP UV-LEDs are more advantageous than classical light sources (xenon and mercury lamps) and helps in the development of a low cost, non-contact, and compact system for continuous real-time fieldwork. This work analyzes the wavelength, output optical power, and the effects of viscosity, temperature of the water pollutants, and the functional consistency for long-term HP UV-LED working operation. To accomplish the latter, an analysis of the influence of two types 365 nm HP UV-LEDs degradation under two continuous real-system working mode conditions was done, by temperature Accelerated Life Tests (ALTs). These tests estimate the mean life under continuous working conditions of 6200 h and for cycled working conditions (30 s ON & 30 s OFF) of 66,000 h, over 7 years of 24/7 operating life of hydrocarbon pollution monitoring. In addition, the durability in the face of the internal and external parameter system variations is evaluated.

## 1. Introduction

Water is one of the most important resources necessary for sustaining life on earth. Population growth and the consequent creation of infrastructures, sewage treatment plants, crops, industries, intensive agriculture, *etc.*, and the associated traffic generated implies a relevant/critical hazard for inland water. In the case of inland water pollution by hydrocarbons and other pollutants which emit fluorescence under high energy light, new developments and analyses are being conducted [1,2,3,4,5,6,7,8,9,10,11,12]. The conventional methods used in recognizing a hydrocarbon pollutant involves transporting a sample from the area of water pollution and identifying the pollutant in the laboratory, most often using the fluorescence spectroscopic method [13,14].While this is an accurate method, it involves too much time for a rapid, and in some cases, programmable automatic action, to mitigate the pollution effect. Therefore, new flexible non-contact and continuous real-time systems need to be developed to identify the hydrocarbons and avoid or mitigate dangerous pollution episodes.

Hydrocarbons are volatile compounds which respond clearly with fluorescence when they are excited with a light source of a specific wavelength range, usually the ultraviolet range, due to its high energy of radiation. This characteristic has been utilized over the decades to identify the hydrocarbon type and its compounds, initially in health applications [15,16], and later in petroleum industrial applications [17]. The basic method involved in fluorescence spectroscopy is to apply an excitation source (light) to the sample and read and analyze the spectra emitted in response [14]. The classical equipment consists of optical, electrical, and mechanical elements, with several alternatives for each, as described in [13]. Light sources include [13]: xenon lamps, high pressure mercury vapor lamps, xenon-mercury arc lamps, lasers, and LEDs (Light Emitting Diodes).

The HP (High Power) UV-LED (Ultraviolet Light Emitting Diode) has significantly increased its optical output power over the last few years, being adopted as an alternative light source for the spectroscopic sensing systems, as it shows some advantages over the classical exciting lights, *viz.*, small size, less cost, quick output optical power stabilization and long life. The UV-LEDs emits in a narrow band of wavelength [18,19,20]. To obtain the fluorescence signature at different wavelengths sources the use of several LEDs are needed [2,3,4], or the combination with other light sources [21]. The classical spectrometer laboratory equipment [14], has been used for decades in the identification of pollutants by fluorescence in a wide wavelength range. However, the employment of LED or UV-LED has enjoyed only a short field experience in the fluoro-sensing applications in real-time [2,3,4,7,8,10]. This work proposes to move a step further from the occasional real-time measurements to continuous real-time detection and pollutant identification.

**Figure 1:**
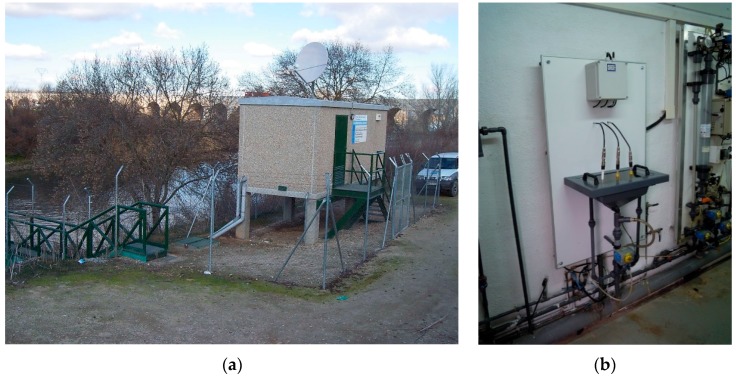
(**a**). Automatic station for recording water parameter measurements; (**b**) The analysis-sink in the automatic station for the multi-parametric meters (pH, conductivity, temperature, and dissolved oxygen) on the Jarama-Tagus Rivers, Spain.

This work studies the functional long-term working of fluoro-sensing using HP UV-LED as the source light, for non-contact pollution detection and identification in inland water, in real-time 24/7 (24 h, 7 days), focusing on hydrocarbon analysis. The future short-term objectives are recognition of the signature pattern and integration of the whole experimental system in automatic water quality station of the Jarama-Tagus Rivers (Spain) [22,23], working 24/7 (Figure 1). In these automatic stations, the authors test different disciplines related to the development of new capabilities, such as the present work, which analyzes the HP UV-LED in the long-term, non-contact, continuous, real-time fluoro-sensing identification of the pollutants [20,24,25], as well as others, like the development of compact, low-power consumption high security warning communications systems with redundant dual-band based mobile phone systems (380 MHz and 960 MHz) [26,27].

In these automatic stations (Figure 1a), the water collected via a pump is conducted to an analysis-sink where the main parameters *viz.*, temperature, conductivity, dissolved oxygen, and pH are measured (Figure 1b). We proposed that the non-contact hydrocarbon identification sensor system would share this installation, and all the parameters measured of the automatic station [12], could be used to improve the identification signature of the hydrocarbon by our fluoro-sensing system by increasing its individual potentialities [28]. At present, the quality parameter measurements can indirectly identify the presence of water pollution, but not the pollutant type. From our experiments, a fluoro-sensing real-time 24/7 pollutant detection and identification system will provide a significant improvement to the existing analyzers in the automatic stations, as it does not involve significant additional cost or maintenance, but it does increase the real-time warning and safety in environmental monitoring.

Furthermore, the spectroscopy workbench instrumentation developed for this HP UV-LED work analysis is in accordance with the new generation of compact, real-time, low cost [7,8], low and easy power, fixed [9] or portable [2,3,12], autonomous systems [4], that connect to a PC or single-board computer. The experiments over the last few years dealt with the detection of substances such as chlorophyll and phytoplankton [2,3,4,7,8,9], besides pollutant detection [6,20].

In this work, we analyzed the suitability of using HP UV-LEDs for long-term continuous detection of the hydrocarbons in real-time utilizing fluoro-sensing. To achieve this, we have described several activities, *viz.*, the method and laboratory workbench, analysis of absorbance and pollutants fluoro-sensing signatures, influence of viscosity and temperature, and processing spectra readout. The final analysis is related with the functional operation and durability for long-term of two 3W 365 nm HP UV-LED technologies, based on the temperature accelerated life tests (ALT) under two different working conditions: continuous and cycled with 30 s ON & 30 s OFF power.

The results and conclusions of this functional analysis are oriented to continuous real-time hydrocarbon detection in automatic river stations; however, they can be essentially extended to other pollutants in water, and other alternative systems either portable or submersible systems.

## 2. Materials and Method

### 2.1. Materials

The hydrocarbons analyzed are the ones commonly used in Europe in gas stations and heating boilers. The samples that were drawn in Spain include two types of gasoline with different octane ratings, two Diesel A types for automotive use (containing different additives, with the ability to cause changes in the spectral response) present in the gas stations, Diesel B for agricultural vehicles and industrial uses, and Diesel C which has other uses including heating boilers because of its better calorific value. The complete list is given below:

- Gasoline 95 and 98 octanes.
- Diesel types A and A Plus, B, and C.

The samples were collected several times during two years from different gas stations of the same multinational European brand, and stored in glass laboratory bottles, inside chamber to keep its temperature at of 15 °C to prevent vaporization due to their high volatility. No differences were observed between the signatures of the samples during these years. The samples were renewed every few months, by to the modification of the signatures due to evaporation in the container, especially in gasolines. Several micro-spectrometer aperture times were tested, and 10 ms with five average measurements was selected as a compromise between number of signature counts and measurement time.

**Figure 2:**
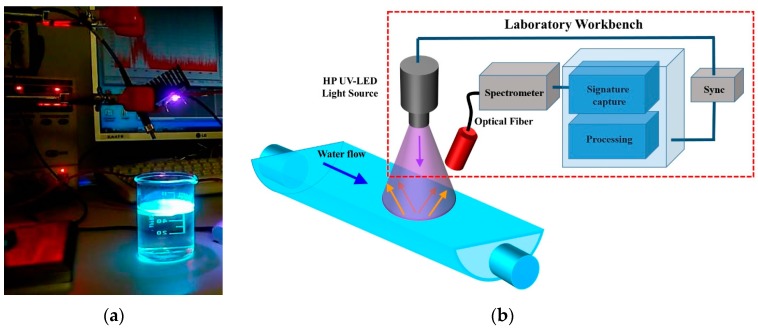
(**a**) Photograph laboratory workbench: optical fiber (left of UV-LED) HP UV-LED and Pyrex beaker containing the fluorescent hydrocarbon; (**b**) Diagrammatic representation of the instrumentation elements of the laboratory workbench.

In order to arrive at a standardized response during the experiments, all the measurements were recorded in a laboratory at 24 °C using a Pyrex beaker filled with water and 1 mm uniform thickness of each different type of hydrocarbon (Figure 2a).

The 1 mm thickness election has been used in a previous work [20] and selected as low limit of identification. Larger thicknesses of hydrocarbon imply higher absolute fluorescence signature values. We suppose that a sample taken in a short distance to source pollution will have a thickness significantly higher than 1 mm. To our knowledge, there is not references related with hydrocarbon equilibrium thickness at long distances in inland water, but in seawater the evaluated hydrocarbon equilibrium thickness at t∞ is between 2 mm and 12.5 mm [29]. Therefore, the selection of 1 mm thickness is a conservative value to identify real hydrocarbon pollution.

### 2.2. Laboratory Workbench Instrumentation

The choice of a non-contact system instead of the peristaltic system employed in the other applications to identify diluted materials [3,4], was because of the future integration of the present workbench instrumentation in the analysis-sink with open fluent water, as shown in Figure 1b. This method is advantageous because it is clean and the LED light analyzes fluent water; however, the drawback is that it requires a higher optical output power because the HP UV-LED needs to be separated from the water in order to avoid the fluent water staining the LED lens.

In Figure 2a a photograph of laboratory workbench instrumentation measurement exciting fluorescent hydrocarbon is shown. In Figure 2b the block diagram representing the laboratory workbench is displayed.

The main elements shown in the laboratory workbench instrumentation block diagram are:

- UV light source, HP UV-LED. A 3W 365 nm LED was chosen for its simplicity and better beam light concentration than a circuit with several LEDs. The HP UV-LED is placed perpendicular at water surface at a distance of 8 cm, the view angle of the UV-LEDs is 2θ_1/2_ = 70°. The optical fiber has an angle of 30° with respect UV-LED perpendicular line, and a 6 cm distance to water surface.
- Spectrometer and optical fiber. Fluorescence detection systems based on compact CCD spectrometer are emerging as a tool in research field applications [2,3,4,20]. In this work two compact spectrometers (Avantes^®^ Ava-Spec 2048-USB2-UA, and Mightex^®^ HRS-BD1-025) have been used, the first one for workbench instrumentation in signature readout, and the second one for analysis of UV-LED temperature ALT [24,25]. The spectra readout is obtained via a specific optical fiber of 0.6 mm Ø, solid angle Ω = 0.15 sr, numerical aperture NA = 0.22 (Figure 2a). Considering the perpendicular distance to the water is 6 cm, the measurement area of fluorescence is 6.73 cm^2^. The wavelengths range of Avantes^®^ Ava-Spec 2048-USB2-UA is 200–1100 nm, and the Mightex^®^ HRS-BD1-025 with wavelengths range 300–1050 nm, proprietary measurement software, power supply, and PC connection by USB cable. This element is the only significant system cost, around 2500$.
- Processor block. This element is implemented in a Windows laptop computer under the software proprietary of Avantes^®^ integrated in MATLAB^®^ (MathWorks^®^, Natick, MA, USA). The micro-spectrometer readout is loaded in a MATLAB^®^ application, which processes the signal for pollutant identification. The objective of this work is to make a qualitative identifications of hydrocarbons in an early warning system, therefore the measurements of the time-dependent decay of fluorescence have not been developed.

The temperature ALT instrumentation is explained in [24] where the instrumentation and preliminary results of ALT are described. The HP UV-LEDs used their characteristics, and electrical and optical operational working conditions are exhaustively described in [25].

## 3. Results

### 3.1. Wavelength Analysis for Hydrocarbon Identification

The introduction of LED as light source for spectrometric instrumentation has increased over the last years, because it is much simpler, it minimizes the instrumentation costs, it has lower power consumption and shows higher reliability. Currently, there are High Power 1–3 W (electrical) UV-LEDs up to 365 nm from different manufacturers (Nichia, Led Engin, LG, Lumileds). Today, this is the working wavelength limit for non-contact high area fluent water fluorescent analysis. Commercial LEDs with wavelength lower than 365 nm LED (Nichia, Crystal, Roithner Lasertechnik, Qphotonics, and LG for 285 nm) involve higher costs and significantly less electrical and optical power, in the order of mW, with low external quantum efficiency, are used for certain scientific applications [30].

**Figure 3:**
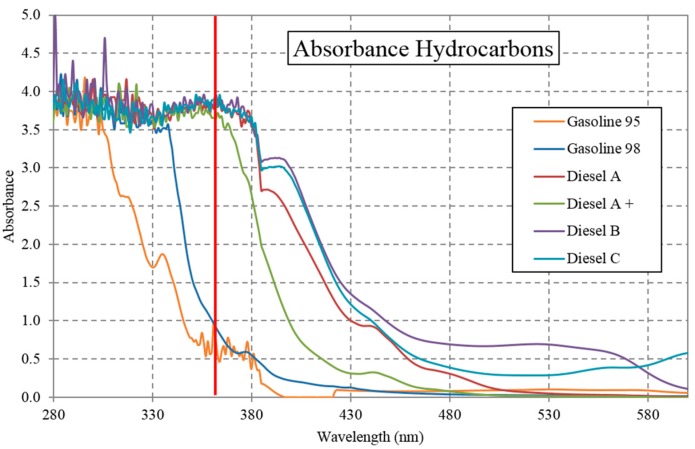
Absorbance of different hydrocarbons compounds used in Europe. Vertical red line at 365 nm.

As the wavelength limitation of HP UV-LEDs is known, we can analyze by absorbance the wavelength range in which the hydrocarbons could emit fluorescence. Absorption spectroscopy is an analytical chemistry tool mainly used to determine the presence of a particular substance [31], but also it contributes to see if the substance could be fluorescent. To produce the fluorescence effect, the substance to be detected must emit at a wavelength greater than the light source used, and in order to emit photons, it must first absorb a part of the light, which usually works closer to ultraviolet [13]. Figure 3 shows the absorbance response for the pollutant substances performed according to criteria ISO/IEC 17025. (General requirements for the competence of testing and calibration laboratories, International Organization for Standardization) using a PerkinElmer's Lambda 25, obtaining the absorbance response shown in Figure 3. This implies that it is possible to determine whether the energy provided by the photons is absorbed by the compound; furthermore, a portion of this energy is emitted as light of a higher wavelength (fluorescence) and another portion of energy as heat in the compound.

**Figure 4:**
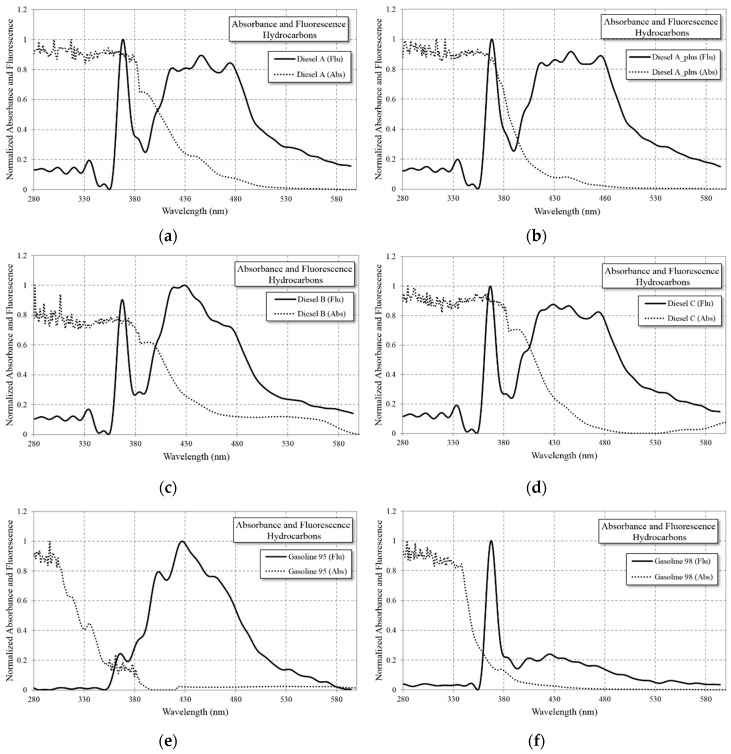
Hydrocarbons normalized fluorescence (continuous line) and absorbance (dotted line) with LED@365nm as the light excitation source. The peaks at 365 nm coinciding with the emission wavelength are due to scattering and reflection. (**a**) Diesel A; (**b**) Diesel A Plus; (**c**) Diesel B; (**d**) Diesel C; (**e**) Gasoline 95; (**f**) Gasoline 98.

In Figure 4, the absorbance curve (dotted line) with HP UV-LED emitted light (3W UV-LED with a narrow peak at 365 nm) and the fluorescence (continuous line) response of the hydrocarbon are shown. The absorbance and fluorescence response are normalized at the maximum count number of the spectrometer readout. From Figure 4, it can be observed that the diesels have higher fluorescence than the gasolines, and the signatures of each hydrocarbon is unequivocal.

These results seem to confirm the relation between absorbance and fluorescence. All the compounds except Gasoline 98 have very high signature levels, as shown later, although in this case, the 365 nm HP UV-LED is sufficient to identify the hydrocarbon; however, a UV-LED source below 365 nm will be much more effective in identifying this hydrocarbon. The limitation of output optical power of UV-LEDs under 365 nm will show significant improvement over the next few years due to the extensive use of UV-LEDs technology, which is continuously and intensively being developed [19].

### 3.2. Viscosity and Temperature Influence in Hydrocarbon Identification

Several factors are found to affect hydrocarbon fluorescence, among which temperature and viscosity are the main variables that can affect the hydrocarbons diluted in inland water. Therefore, it is necessary to quantify the impact of these factors on the spectrometer readout results, in order to consider this in the signature identification.

Temperature can decrease the fluorescence power between 1% and 5% per degree Celsius increase. As the temperature increases, the viscosity decreases and the frequency of collisions increases, increasing the probability of deactivation as non-radiant energy. In the case of hydrocarbons, it is necessary to measure the change in viscosity with temperature, although it is expected that because these compounds are highly volatile, they naturally have very low viscosity.

**Figure 5:**
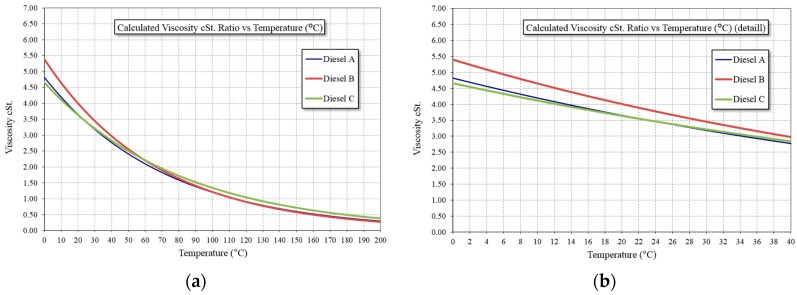
Viscosity measures of Diesel types in two temperature ranges. (**a**) Wide temperature range; (**b**) Detail of temperature range in inland waters.

In Figure 5a, the viscosity measurements are shown for three types of fuel, observing no great difference among them in the whole temperature range. Measurements are performed according to ASTM D-445 (Standard Test Method for Kinematic Viscosity of Transparent and Opaque Liquids (and Calculation of Dynamic Viscosity), ASTM International). Details regarding the temperature range at which they can occur in water are shown in Figure 5b in which the viscosity variations are between 3 and 5 cSt.

To corroborate the results presented, some fluorescence measurements taken using UV-LED as the light source, recorded under the same conditions at different temperatures, clearly highlight the influence of temperature on fluorescence. The temperatures selected are 5, 21, and 35 °C, values that can be observed in different field conditions.

**Figure 6:**
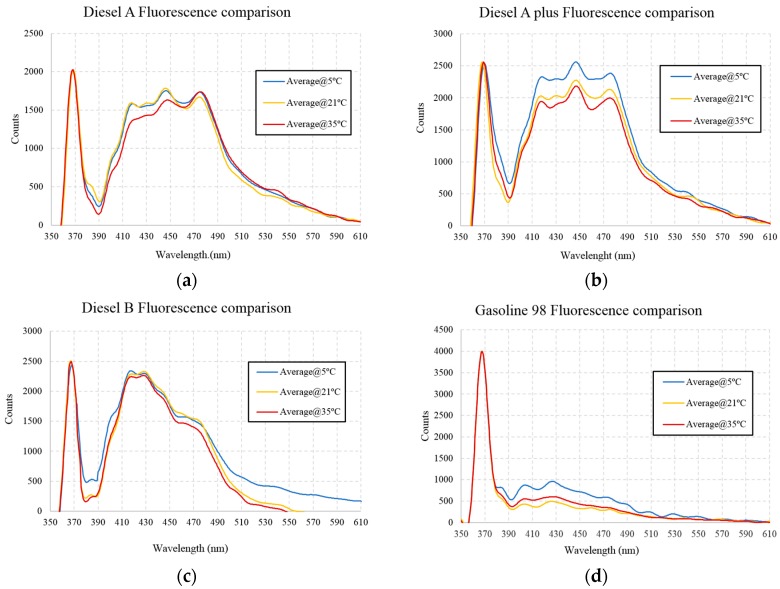
Fluorescence of the hydrocarbons at different temperatures with LED@365nm as the light source. (**a**) Diesel A; (**b**) Diesel A Plus; (**c**) Diesel B; (**d**) Gasoline 98 octane.

In Figure 6, four representative hydrocarbons are shown as examples. As evident from the results, at lower temperatures a greater fluorescence response is obtained. However, from Figure 5, two different cases can be observed:

- In the case of Diesel A, the response at 35 °C is different from the others at lower temperatures because the fluorescence peak at 425 nm is lower at 35 °C. This difference cannot be corrected with a normalization of the signal because it depends on the wavelength, in this case a different master signature for low, medium, and high temperatures are required, and furthermore, a real-time measured temperature is needed for a correct identification of the signature [12,28].
- In the case of Diesel A Plus and Gasoline 98 octane, the difference in the optical power between the high and low temperatures is much greater than for the other hydrocarbons.

The main conclusion drawn is that if, during a year, extreme temperature ranges occur in the river waters, a complementary temperature measurement is needed in order to identify a direct relationship between the fluorescence response and the pollutant.

### 3.3. Processing of Spectra Readout

The readout of the CCD micro-spectrometer has two components; reflected UV-LED signature and significant fluorescence signature. The UV-LED spectrum is represented in Figure 7 of [25], observing a very low optical power of UV-LED at wavelengths higher than 400 nm, for that, we considered the significant fluorescence signature in the range between 400 nm and 650 nm. Over 650 nm, the fluorescence signature CCD counts are near to zero.

**Figure 7:**
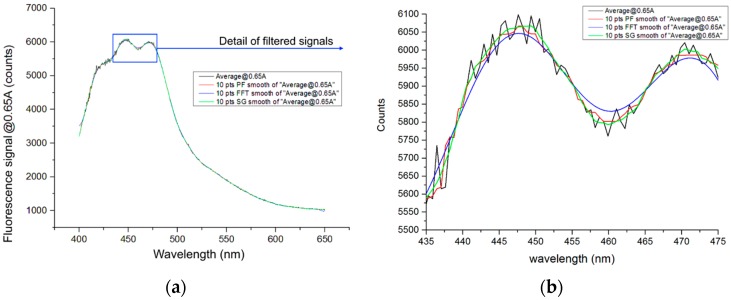
(**a**) Different filtering methods tested for significance of fluorescence; (**b**) The detail of the signatures are shown in black line readout, red line median smooth, blue line Fast Fourier time filter, and green line Savitzky-Golay smooth.

The second step involves reducing the noise and improving the signature, and several filter types have been tested, between themselves: median smooth, Fast Fourier Time, and Savitzky-Golay (S-G) smooth. Figure 7 shows the results for Diesel A in the UV-LED normal working condition.

**Figure 8:**
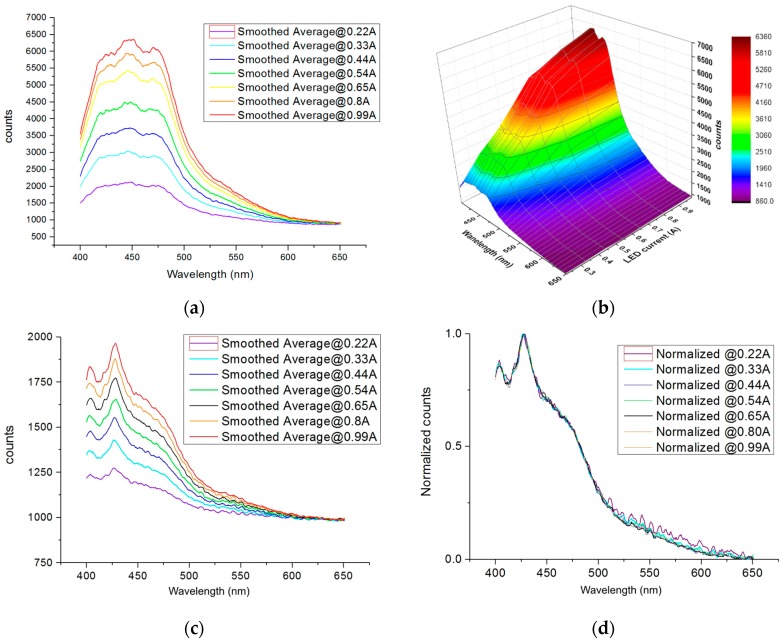
(**a**) Fluorescence signatures after the signal was processed without normalization of Diesel A Plus at different current supplies; (**b**) 3D fluorescence graphic for Diesel A Plus; (**c**) Fluorescence signatures after the signal was processed without normalization of Gasoline 98; (**d**) Fluorescence signatures processed with normalization for Gasoline 98.

The Avantes^®^ micro-spectrometer as shown in Figure 7a and Figure 8c set the minimum count value in 1000 counts; after filtering the signal it is normalized to the maximum value between 400 and 650 nm. The final results are shown in Figure 8d. This process, reduces a great part of the changes in the readout due to the UV-LED different working conditions; ambient temperature, LED temperature, situation of the LED optical output power degradation in the long-term, *etc.*, producing a significant improvement for signature identification.

As evident in Figure 7b, a detail of the S-G smooth filter, widely used in the chemometrics spectrometer analysis, reveals the best results [32,33,34], it does not reduce the peaks and increase valleys of the fluorescence (Fast Fourier time filter) and does not modify the position of the wavelength peaks; furthermore, it shows the best smooth as the median smooth. In conclusion, an S-G filter with 10 samples (one sample each 0.589 nm) and polynomial of 2 degrees is elected for processing all the readout signatures, post normalization

### 3.4. Influence of HP UV-LED Parameters in the Spectrometer Readout

The HP UV-LEDs are more advantageous than the lamps, but is a less mature technology and currently undergoing development of materials and processes [18]. HP UV-LEDs are designed for working over a range of current supply, the voltage level is defined by the defined current supply by means of the I-V curve, and the output optical power of LED, an increase in the current (power supply) of UV-LEDs supposes an increase in the optical output power. However, an increase in the current supply implies a higher current stress and temperature which affect the LED efficiency and reliability. In Figure 8, the fluorescence response is represented for the different currents, which implies different outputs of optical power. Furthermore, the high current stress of the HP UV-LED (tested at 3W UV-LEDs over a current density of 60 A/cm^2^) makes it necessary to have a large sink to avoid the high semiconductor junction temperature. Both HP UV-LEDs types have high thermal conductivity ceramic encapsulation and with one unique chip. The description of the complete package and thermal state of the HP UV-LEDs under test are given in [25].

During long-term operations, the LEDs suffer degradation in the semiconductor and encapsulation materials which affect the LEDs efficiency. As it is not a mature technology, UV-LEDs of the same type have similar characteristics but show differences for the same binning in optical power of 5%–10% among them. Therefore, it is necessary to record the fluorescence master signature prior to normalization in the normal working conditions for a typical HP UV-LED emission of one type, as it cannot be the same for the other UV-LEDs, these variations will be considered in the analysis [35].Thus, it is necessary to consider these optical output power variations, the long-term output optical power degradation, and other negative effects as due to the cloudiness of the LED lens. As these parameters influence and affect the optical output power in a similar way to the current, and the influence of currents on fluorescence readout are analyzed. To achieve this, a new 3W HP UV-LED was supplied with power of different current levels, emulating the different states of degradation during life. 

The normal working condition defined for the two technologies (A and B) of 3W HP UV-LEDs are 600 mA [25], that is 85% of the 700 mA defined as the nominal and maximum value for the continuous working condition according to the manufacturers in the datasheet, other parameters of two LEDs types appear in Table 1. Voltage is defined for the characteristic I-V curve of the HP UV-LED. The optical output power of both the UV-LEDs types under test are similar, with a difference of over 15%.

For a conservative election, we measure the signature with the UV-LED type of minor output optical power. The relation between current applied and output optical power measured at 8 cm for 600 mA, with Juno-USB equipment and 3A optical sensor, both of Ophir^®^, is 9.11 mW/cm^2^, for other supply conditions used in fluorescence characterization the values are: 14.45 mW/cm^2^ at 0.99 A, 11.92 mW/cm^2^ at 800 mA, 9.86 mW/cm^2^ at 650 mA, 8.26 mW/cm^2^ at 540 mA, 6.68 mW/cm^2^ at 440 mA, 4.97 mW/cm^2^ at 330 mA, and 3.24 mW/cm^2^ at 220 mA. As it can be seen, the light output is not proportional to current, the best relation is over the 0.6A, and it is worse at higher currents with elevated temperatures, this also is reflected in the fluorescence measured in hydrocarbons Figure 8b.

In Figure 8, it is evident that the signatures of the two hydrocarbons are different at different HP UV-LEDs power supplies (current injections). The hydrocarbons selected are: Diesel A Plus that has a good fluorescence spectrum and shows the highest values, whereas Gasoline 98 with a relatively poor fluorescence level shows the lowest values. In the case of gasoline, the relation between signal and noise is minor, and recognition is more difficult at low currents of operation, which emulates a higher level of degradation; for example, the readout at 440 mA is equivalent at a maintained optical power output of 73% compared with the initial nominal condition (6.68*100/9.11 = 73%), at 330 mA it is 54.44% and at 220 mA it is 25.6%.

In order to define a level of optical output power degradation that enables the functionality of the HP UV-LED for identification of fluorescence pollution several issues have been considered:

- The variations in the initial optical outputs of power of the new LEDs identified earlier.
- The optical output power degradation during the life of the LED, and the additional optical output power losses during real operation due to a dirty lens or dust.
- A 30% optical output power degradation with respect to the initial optical output power value is the standard definition of degradation failure in white illumination LEDs [36]. The reliability datasheets of the LEDs that include the standard report IES-LM-80-08 (Approved Method for Measuring Lumen Maintenance of LED Light Sources) [37] also select this failure limit evaluating the MTTF_(L70)_ (Mean Time to Failure assuming failure when optical output power decays below 70% of the initial optical output power). The other, less frequent failure modes of the LED are the catastrophic failures due to short or open circuit. During temperature ALT, no catastrophic failures have been observed, all detected failures are due to light output degradation over 30%.
- In Figure 8d, the normalized signatures in the worst fluorescence signature case (Gasoline 98), for different currents have been represented. The signature at 220 mA (violet) has been observed to have higher noise than the fluorescence curve at nominal current (650 mA black line), the 330 mA (cyan) fluorescence signature (with a 54.4% of the optical output power with respect to the nominal current) has significantly less noise and therefore, we will consider that 220 mA is not a functional power at which to identify Gasoline 98. Therefore, although 50% or 60% of the initial optical output power would suffice, considering the other possible factors of optical output power that have been explained in the prior paragraph, we conservatively selected 70% of the initial optical output power, which is the standard value [36] and it is contemplated in the reliability datasheet of the commercial LEDs.

### 3.5. Long Life UV-LED Test for Hydrocarbons Detection

ALT (Accelerated Life Test) is an alternative method to assess the life of a device in a short period of time. Temperature ALT that offers an estimation of the total life in some thousands of hours has been developed in order to assess the life of the HP UV-LEDs in order to identify the hydrocarbons.

**Figure 9:**
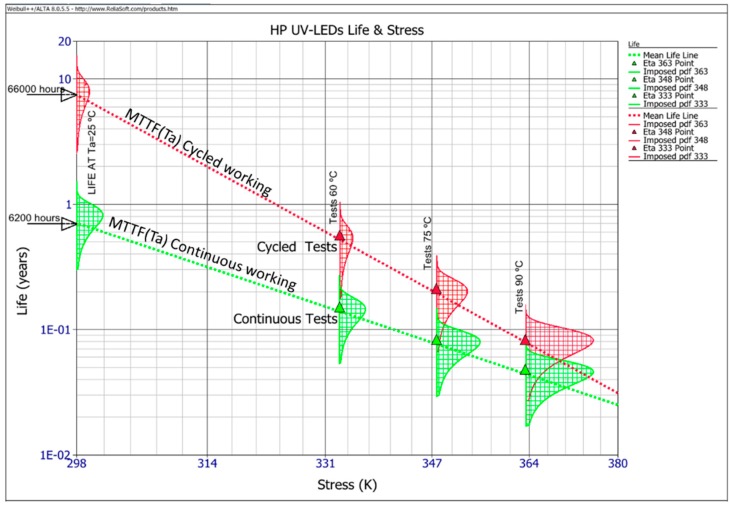
Evolution of the HP UV-LEDs life *vs.* ambient temperature (Ta) stress. The continuous line represents failure density probability function, the dotted line MTTF_(L70)_(Ta) shows continuous (green) working and cycled (red) working (30 s ON & 30 s OFF).

This acceleration of time working is based in that the catastrophic or degradation failure mechanisms of LED technology is accelerated by temperature, and this time acceleration is modeled physically and mathematically by the Arrhenius model [38]. This model is the general application used in LEDs by manufacturers [39,40], with standardization of degradation life prediction IES-TM-21-11 (Projecting Long Term Lumen Maintenance of LED Light Sources) [41], and research reliability of the III-V optical semiconductors devices [42,43,44,45,46]. By this model, the failure distribution of the HP UV-LED population under test at a nominal temperature of 25 °C, is reproduced in accelerated time by the Arrhenius model at higher temperatures. To achieve this, the process involved estimating the complete life evolution of a population of LEDs in nominal working conditions, involving the inverse process, to develop accelerated tests at higher ambient temperatures, in this case 60 °C, 75 °C, and 90 °C at the same nominal working conditions of the HP UV-LEDs and characterizing periodically the electrical, thermal, and output optical power parameters during the tests [25], for final analysis (Figure 9).

In this case, we estimate the life of two types of 3W 365nm HP UV-LEDs. For temperature ALTs three ovens with three (Ta) ambient temperatures (60 °C, 75 °C, 90 °C) were used during 4000 h for each test. The LEDs have been tested under two different working modes at 600 mA; continuous and cycled (30 s ON & 30 s OFF) completely analyzed in [25].

During ALT, we measured the instant time of degradation failure (70% of the initial power) for the LEDs with high degradation, and the extrapolation of this time for those LEDs that did not get degraded below 70% initial power during the temperature ALT. With the failure times of the LEDs in all the experiments (three temperatures and two working modes) the results were analyzed, applying the Arrhenius model. To accomplish this, we employed the specific software for the temperature ALT reliability experiments, Weibull++/ALTA of Reliasoft^®^ (ALTA—Accelerated Life Test Analysis application) [47].

The complete results by Weibul++/ALTA obtained are shown in Figure 9, in the continuous working mode (green color) and cycled working mode (red color) for the HP UV-LED type with the higher MTTF_(L70)_ (25 °C), Mean Time To Failure at Ta = 25 °C and 70% limit of maintaining light output. This Figure shows the failure distribution of the population under test (Figure 9, red and green vertical draw functions), at 60 °C, 75 °C, 90 °C Ta, and the extrapolation by the Arrhenius model at the nominal Ta = 25 °C. In Figure 9, representations were also done by the red and green continuous lines of MTTF_(L70)_(Ta) at each temperature. For the other type of HP UV-LED under test, the results for continuous working are very similar, and sensibly minor for the cycled condition, but in any case, several times better than for continuous working. Other relevant aspects during the tests related with the failure analysis are the degradation of the silicone encapsulation of the UV-LEDs, a slow progressive degradation of the optical output power, and a slight peak shift to the higher wavelengths (1.5 nm), that are described in [25].

The MTTF_(L70)_ obtained for the HP UV-LED type with the longest life at 25 °C ambient temperature, is 66,000 h (seven-and-a-half years of working life) for cycled working conditions 30 s ON & 30 s OFF [25] and 6200 h for the continuous mode (eight-and-a-half months of working life). For the other technology, HP UV-LED, in cycled working mode the MTTF_(L70)_ was over 2,9000 h, and 5700 h for the continuous working mode.

The results of ALT enable the following conclusions:

- For the continuous working mode HP UV-LEDs MTTF life of eight-and-a-half months, is better than or similar to continuously working spectrometric lamps.
- For the cycled mode, the MTTF life of more than seven years for one type of the HP-UV-LEDs and four years for the other type, are considerably better than that of the conventional spectrometric lamps in the continuous operation mode. It must be considered that the conventional UV-lamps cannot operate in the cycled (1 min) mode, due to the great stabilization times.
- The continuous working mode has a short life compared with the cycled working condition, where for 30 s of each minute the water pollutants are not sensed. A partial solution will be to switch the power supply every 30 s to two different LEDs to excite the fluorescence, and although the time for stabilizing the optical output power is over 7 s and the cost of each 3W HP UV-LEDs is roughly $35.

We have also compared, after ALT, the LEDs that have been degraded with the brand new LEDs working at different currents based on power supply. The results are consistent with those recorded in Section 3.4, observing the good quality of fluorescence pollutant detection when the optical output power drops below 30%. To accomplish this, we proposed as the failure degradation limit a conservative value of 70% of the initial optical output power.

The process involved in obtaining these results has been extended over time: the authors studied the HP UV-LEDs with respect to the hydrocarbons comparing them with other classical light sources in [20], and later developed a series of experiments with two types of new commercial technologies 3W 365 nm HP UV-LEDs, characterizing the encapsulating, thermal, and electrical response in two working modes: continuous and cycled (30 s ON & 30 s OFF) [25], indicating the method and the preliminary results of the ALT [24,25] with these two types of UV-LEDs and the two working conditions in [24].

## 4. Conclusions

The main objective of this work is to validate the continuous real-time detection and identification of the hydrocarbon pollutant, during the long-term operation of the HP UV-LEDs as the excitation light of the fluoro-sensing systems. Extrapolation of these results for any pollutant detected using a similar real-time technology is relevant, because any improvement in the reaction time in a water pollution accident could be critical.

The main conclusions drawn from this work are given below:

- The materials, process, and laboratory equipment workbench used have been discussed. The detection system proposed has been performed without any contact with the fluent water, which offers several advantages, as it avoids soiling of the intermediate elements (glass, lens) of UV-LED while it analyzes a large area. The sensitivity and detection area could be improved by reducing optical losses and increasing the solid angle for fluorescence detection, using a lens or lens system.
- The absorbance and fluorescence signature of the different hydrocarbons have been analyzed using the selected HP UV-LED, and the influence of temperature and viscosity in the signature have been studied.
- After testing several types of signal processing, it has been proposed that a Savitzky-Golay filter and signal normalization in the range of 400 to 650 were the best method to obtain a repetitive signature under different conditions of the parameters, including the possible optical output power degradation of the HP UV-LED.
- The influence of HP UV-LED long-term output optical power degradation by current supply emulation, in fluorescence signature detection error was analyzed and the degradation limit of 30% was noted. Then maintaining 70% of the initial optical output power of the HP UV-LED was selected as the conservative limit for the repetitive identification of all the hydrocarbon signatures. Degraded LEDs in the ALT with optical output power degradations below 30% value show an optimum fluorescence signature.
- In order to validate the long-term life of the HP UV-LEDs, temperature ALTs have been developed. The complete setup of the parameters in this test are explained in [24,25], and it has been assessed and extrapolation of failure distribution is shown (Figure 9). From these data, it can be concluded that the MTTF_(L70)_ for the cycled working mode (30 s ON & 30 s OFF) is several times better than that obtained for the continuous working mode.
- For long term operation, the HP UV-LEDS with continuous working mode has an estimated MTTF_(L70)_ ≈ 6200 h working, and for cycled working it depends on the type technology of LED, MTTF_(L70)_ ≈ 66,000 h for HP UV-LED with long life type. In all cases, the results of MTTF_(L70)_ are very promising, mainly for the cycled 30s ON & 30s OFF working condition.

As the technology of HP UV-LED is very innovative, new generations of UV-LEDs will significantly improve. In this work, we have validated HP UV-LEDs as excitation light sources of non-contact, continuous real-time fluoro-sensing pollutants for long-term applications, thus, these results open up new possibilities in fluoro-sensing.

At the current state, we have probed the viability of detecting saturated hydrocarbons in a laboratory environment, in order to transfer this technology to real conditions, it will be necessary to analyze the hydrocarbons signatures influenced by real samples of river water, with different aqueous matrices (e.g., various organics, biomolecules, nutrients, *etc.* which are in water miscible/soluble). Other future work is related with the identification of mixtures of hydrocarbons in different proportions, emphasizing the hydrocarbons mixed with car oil, since it is a standard type of pollution.

## Figures and Tables

**Table 1:** Main electrical and optical parameter of HP UV-LEDs at nominal and at working condition.

HP UV-LED Type	Nominal Electrical/Optical Power (W)	Electrical/Optical Power at 600 mA (W)	Irradiance at 8 cm & 600 mA (mW/cm^2^)
A	≈3/0.8	≈2.4/0.65	9.11
B	≈2.8/1	≈2.25/0.75	10.40
